# Human Papilloma Virus Associated Squamous Cell Carcinoma of the Head and Neck

**DOI:** 10.1155/2015/791024

**Published:** 2015-09-21

**Authors:** Vidya Ajila, Harish Shetty, Subhas Babu, Veena Shetty, Shruthi Hegde

**Affiliations:** ^1^Department of Oral Medicine and Radiology, A B Shetty Memorial Institute of Dental Sciences, Nitte University, Deralakatte, Mangalore 575018, India; ^2^Department of Ophthalmology, K S Hegde Medical Academy, Nitte University, Deralakatte, Mangalore 575018, India; ^3^Department of Microbiology, K S Hegde Medical Academy, Nitte University, Deralakatte, Mangalore 575018, India

## Abstract

Oral cancer is one of the commonest causes for mortality and morbidity with squamous cell carcinoma being the sixth most frequent malignant tumour worldwide. In addition to tobacco and alcohol, human papilloma virus (HPV) is associated with a proportion of head and neck cancers. As in cervical cancers, HPV types 16 and 18 are the cause of malignant transformation. HPV-positive cancers of head and neck have unique characteristics such as occurrence in a younger age group, distinct clinical and molecular features, and better prognosis as compared to HPV-negative carcinomas. They also possess the potential for prevention by using vaccination. The present review describes in detail the salient features of HPV associated oral squamous cell carcinoma (OSCC), its differences from HPV-negative OSCC, diagnostic features, and recent strategies in prevention and management.

## 1. Introduction

Oral cancer has been defined as “a neoplasm involving the oral cavity which begins at the lip and ends at the anterior pillar of the fauces” [1]. Squamous cell carcinoma (SCC) is the most frequent oral cavity malignancy accounting for over 90% of oral cancers [2]. It is defined as “a malignant epithelial neoplasm exhibiting squamous differentiation as characterised by the formation of keratin and/or the presence of intercellular bridges” [3]. It represents the sixth most frequent malignant tumour worldwide [2]. Human papillomavirus (HPV) is the commonest sexually transmitted viral infection [4]. HPV has also been established as the causative agent in cervical cancer.

Commonly associated with benign skin and oral lesions, recent reports suggest the involvement of HPV in oral and oropharyngeal cancers [5]. 

*Structure of HPV*. HPV belongs to the family Papillomaviridae and is a small, nonenveloped DNA virus having diameter of 52–55 nm. The genome consists of double-stranded DNA bound to cellular histones and surrounded by a protein capsid [4]. The eight open reading frames (ORFs) have three functional components: the early (E) region, the late (L) region, and a long control region (LCR). The early region is essential for replication, cellular transformation, and viral transcription; the late region forms structural proteins (L1-L2) essential for virion assembly; and the long control region is necessary for the replication and transcription of viral DNA [4].

## 2. History

zur Hausen, in the 1970s, proposed that cervical cancer and condylomata acuminate may share a common viral etiology, that is, HPV 6–8 [6]. Syrjanen in 1983 first suggested HPV as a causative agent of head and neck cancer due to the similar clinical features in oral and genital injuries as well as similarities in the epithelia, affinity of HPV for epithelial cells, and its oncogenic potential [7].

## 3. Epidemiology

A systematic review of HPV associated head and neck squamous cell carcinoma (HNSCC) found a prevalence of 25.9%. The prevalence was higher in oropharyngeal squamous cell carcinoma (OPSCC) (35.6%) compared to oral (23.5%) and laryngeal (24.0%) SCC [8]. The ethnicity and geographic origin of patients are known to be responsible for differences in HPV prevalence in HNSCC. Asiatic countries, particularly Japan, have the highest worldwide frequency. This high prevalence of HPV in Asiatic patients with oral cancers implies that viral infection may be an important etiological agent and along with dietary habits and a probable genetic predisposition can cause additional mutations leading to malignancy. The lowest prevalence of HPV-positive HNSCC was in Africa [9]. Kulkarni et al. [10] in a study conducted in Karnataka, India, found that 96% of cervical cancer was positive for HPV, 70.59% was positive in OSCC, and 84% in general population. Their results showed higher HPV 18 prevalence as compared to HPV 16 in general population [10]. Elango et al. [11] found that 48% of tongue OSCC was positive for HPV 16 using PCR.

## 4. Risk Factors

Oropharyngeal cancers associated with HPV have risk factors such as more number of sexual partners, oral-genital sex, and oral-anal sex. Marijuana use is an independent risk factor for HPV-positive HNSCC, and the risk increases with the intensity, duration, and cumulative years of marijuana smoking.

Marijuana use may have a role in oropharyngeal cancers (OPCs) due to its immuno modulatory effect. Cannabinoids bind to the CB2 receptor of immune-modulatory cells in tonsillar tissue. This causes decreased immune response, reduced resistance to viral infections, and decreased antitumour activity [12]. Toombak, a form of snuff, is associated with oral cancer in Sudan. They found that 46% of oral squamous carcinomas were positive for HPV [13].

Herrero et al. [14] found that HPV is detected less frequently in ex-smokers, current smokers, and tobacco chewers as compared to nonsmokers and nonchewers. HPV detection is also more frequent in subjects with greater than one lifetime sexual partner and in subjects with history of oral sex. The relationship of HPV DNA with sexual behaviour was similar for both oral and oropharyngeal cancers [14]. Ho et al. [15] found that HPV type affected the risk of cervical intraepithelial neoplasia more than the viral load.

## 5. Concordant HPV Types in Saliva and Cervix

Du et al. [16] examined the HPV prevalence in the saliva and cervix of youth in Sweden. Their results showed that oral infection was more frequent in women with cervical HPV infection, although the number of HPV types was fewer. They also found that oral HPV types were completely concordant with cervical HPV types but the reverse was not true. HPV 16 was the commonest type. However, in a study conducted on pregnant women, only 1% had simultaneous HPV infection in the oral cavity and cervix but no cases had similar HPV type [17]. Adamopoulou et al. [18] found that women having cervical HPV infection had more chance of asymptomatic oral infection. A study on HIV positive women found that oral HPV infections were less common than cervical infection in both HIV positive and HIV negative women [19]. The concordance between oral and cervical HPV was low [19]. In Malaysia, low prevalence of oral HR-HPV was seen in women with cervical cancer [20].

## 6. Etiopathogenesis

The oncogenic potential of HPV is because of its ability to incorporate E6 and E7 into the host genome leading to inactivation of the tumour suppressor genes p53 and p16 [9]. E6 protein of HPV can affect p53 in the following two ways: it can bind to it causing its degradation or it can inhibit p300 mediated p53 acetylation which affects its function [21].

Dai et al. [22] found an inverse relation between TP53 mutations and HPV-positive cancers. They also found that TP53 mutations were more common in smokers as compared to nonsmokers and HPV 16 DNA was more frequent in nonsmokers as compared to smokers [22]. HPV mediated tumourigenesis was found to be higher in people with codon 72 Arg/Arg and Arg/Pro status when compared to Pro/Pro homozygotes taken as reference genotype [21]. E7 affects the tumour suppressor retinoblastoma protein (pRb), prevents it from binding to the E2F transcription factor, and thus prevents cell cycle progression. This functional inactivation of pRb leads to increase in p16 tumour suppressor protein p16INK4A. Most HPV-positive HNSCCs show p16 overexpression [6]. The association of HPV and smoking in carcinogenesis is not clear. However, one study stated that low-risk HPV infections may act along with tobacco and alcohol which are known causes of oral and oropharyngeal cancer [23].

The carcinogenic effect of alcohol, tobacco, and other related carcinogens may be increased by HPV infection. Defective apoptosis, neovascularisation, and cellular immortality are also predominantly associated with HPV infection. Thus, HPV mediated carcinogenesis may be an integrated process due to the interplay of various factors. This led to the hypothesis of “a condemned mucosa syndrome” occurring due to viral infection and the associated cellular as well as genetic changes [24].

## 7. Oral and Oropharyngeal Cancer

The International Agency for Research on Cancer (IARC) conducted a multicenter case-control study of oral and oropharyngeal cancer in nine countries. They found HPV DNA in biopsy tissue of 3.9% of oral cavity cancers as against 18.3% of oropharyngeal cancers. HPV 16 was the commonest type (89.3%) [14]. The effect of tobacco and HPV together was evaluated using HPV16 L1 VLPs and antibodies against HPV16 E6 and E7. HPV appeared to be a contributing factor to cancer in smokers and chewers [14]. HPV 16 and 18 are implicated in almost all HPV-positive cancers [25].

## 8. Clinical Aspects

HPV-positive oral cancer occurs in younger age group as compared to HPV-negative cancers with an average age difference of 4–10 years. HPV-positive patients also have higher income and more years of education [26]. The male : female ratio is 5 : 1. HPV associated cancers usually present with smaller primary tumours but more advanced nodal stage [26]. Cystic metastatic neck nodes have been reported and sometimes the tumour can present as metastatic nodal disease with unknown primary [26].

## 9. Diagnosis

The common methods being used in diagnosis of HPV are viral DNA detection with polymerase chain reaction (PCR) or In Situ Hybridization and p16 detection by immunohistochemistry. Amplification of target DNA sequences by PCR followed by hybridization with dedicated probes is the commonest method for HPV detection and genotyping [27].

A recent study used three methods together to determine the HPV influence on occurrence of HNSCCs. They used a combination of p16 immunohistochemistry, Consensus PCR HPVDNA, and In Situ Hybridization to better identify HNSCCs caused by HPV. All the HNSCCs which were HPV-positive by PCR and/or ISH were also p16 positive by immunohistochemistry (IHC), with IHC showing a very high sensitivity as single test (100% in both OSCC and OPSCC) but lower specificity (74% in OSCC and 93% in OPSCC) [28].

Studies have suggested that p16 positivity can be used as a biomarker for HPV associated tumours and also as a prognostic factor in HNSCC [8]. Chaudhary et al. [29] compared the efficacy of PCR and Hybrid Capture II in HPV detection. In OSMF, 86% concordant results were obtained. The sensitivity and specificity of the test were 73.7% and 92.5% for HC II and PCR, respectively, and in OSCC, the results had an agreement of 88.3% with a sensitivity and specificity of 87.14% and 92.76% [30].

Antibodies to HPV can be detected in the serum and is a measure of the cumulative exposure of an individual to HPV infection [30]. Antibodies against HPV16 L1 and E6 were found more frequently in oropharyngeal cancers as compared to oral cancers. Antibodies against HPV16 E6 or E7 were found in 65% of patients with HPV16 positive cancers of the oropharynx. Thus, these can be used as markers of HPV in the absence of appropriate cytologic or histologic specimens [14]. Cameron et al. have used saliva instead of serum to determine HPV antibodies, predominantly IgG, in HIV positive individuals. Saliva was used due to its ease of collection in both pediatric and geriatric age groups, noninvasive methods of collection, and comparable results [30]. However, antibodies obtained through oral sampling are fewer than those in serum; therefore, false negatives may be common [30]. Schwartz et al. [31] found that E6 antibody response was seen seven times more in cases with HPV 16 DNA.

HPV DNA detection is low in exfoliated cells and saliva as compared to tissue. Immune response decreases in HIV patients and HPV detection rates are said to be higher. Adamopoulou et al. [32] found that HIV positive individuals have a threefold higher incidence of HPV positivity which is associated with increased cervical and oral intraepithelial neoplasms [32].

## 10. Histopathology

The pathologic features of HPV-positive tumours are different from HPV-negative tumours in the following:

- They are not associated with surface dysplasia or keratinisation.
- They exhibit lobular growth.
- They have infiltrating lymphocytes.
- Basaloid variants are common.

HPV-positive tumours are usually well differentiated and the basaloid tumours have good prognosis [12].

## 11. Prognosis

Oral squamous cell carcinoma has significant mortality and morbidity rates despite the enormous amount of research in the subject [33]. Higher mortality rates and second primary tumours are more common in smokers, alcohol drinkers, and quid chewers [33]. Cessation of smoking and alcohol habits has a favorable prognosis on OSCC and fruits and vegetables have a protective effect especially citrus fruits, fresh tomatoes, green peppers, and carrots [34]. Low socioeconomic status and education, delay in diagnosis, and comorbid conditions like systemic diseases can affect OSCC outcomes. Rich vascular and lymphatic network, local extension, stage of the tumour at presentation, and cervical node metastasis are associated with poor prognosis [33]. Tumour thickness greater than 5 mm is strongly associated with occult nodal metastases and some authors believe this is superior to TNM staging in determining prognosis. Extracapsular spread, perineural invasion, tumour differentiation, and angiogenesis have shown a role in determining prognosis [33].

Among the molecular markers, aberrations in chromosomes 3, 9, 11, 13, and 17 and tumour suppressor genes like p53 and pRb are implicated [33]. Cytokeratin 8/18 is an independent factor associated with poor prognosis as is abnormal DNA content [33, 34]. Treatment related factors include cervical node dissection and achievement of disease-free margins [33]. Local recurrence rate ranges from 64 to 84% in case of positive surgical margins [34]. The 5-year survival rate in early stage for patients undergoing surgery is 92%, radiation therapy is 69%, and combination therapy is 71%. In advanced cases, this changes to 74%, 37%, and 51%, respectively [34]. Liang et al. [35] found that, among the various biomarkers, HPV16 E6 and E7 in serum were the most predictive in determining the prognosis of HNSCC. They found that patients with p16 +/E6-/E7- had increased risk of death. Patients whose tumours were HPV16 DNA positive but E6 and/or E7 seronegative had no decrease in overall mortality but patients with E6 and/or E7 seropositivity showed significant reduction in mortality irrespective of DNA status [35]. In HPV-positive disease, tobacco usage is an independent prognostic factor; improved prognosis is seen in males but females show no change in survival estimates [26].

## 12. Nutrition and HPV

Ascorbic acid, b-carotene, and a-tocopherol are believed to have a protective effect. Ho et al. [15] found that HPV-positive individuals with plasma reduced ascorbic acid of more than 0.803 mg/dL had decreased risk for cervical intraepithelial neoplasia (CIN) compared to those with less than 0.803 mg/dL. Ascorbic acid has prevented oncogenic transformation in vitro and has suppressed HIV replication in T lymphocytes. Thus, its supplementation may have a role in prevention [15]. A few studies have shown inverse relation between intake of dark green and yellow vegetables, b-carotene, and vitamins C and E with risk of CIN and cancer. Giuliano et al. [36] showed that dietary intake of lutein/zeaxanthin, b-cryptoxanthin, and vitamin C can cause reduced risk of type specific, persistent HPV infection. Intake of papaya, a rich source of dietary carotenoids, was associated with decrease in risk of persistent infection. The protective effect of these nutrients may be due to their activity as antioxidants. They may also be implicated in decreased viral replication and expression [36].

## 13. Vaccination

One of the methods proposed for prevention of HPV related oropharyngeal cancer (OPC) is vaccination. At present, there are two vaccines available: the HPV 16/18 vaccine Cervarix and the HPV 6/11/16/18 vaccine Gardasil [25]. The CDCs Advisory committee on immunisation practices (ACIP) has recommended HPV vaccination for females and males between the ages of 11 and 12 years, as early as 9 years, and booster doses up to 26 and 21 years for females and males, respectively [25]. In order for the vaccination to have a role in prevention of oropharyngeal cancer, the protection needs to last at least for two decades and ongoing studies have shown no waning of systemic antibodies at 8 years after vaccination [25].

## 14. Management of HPV-Positive Head and Neck Cancer

The improved survival outcomes have led to the investigation of reduced radiation dose in HPV-positive head and neck cancers [37]. Ongoing trials include induction chemotherapy with response adapted radiation, alternatives to cisplatin given concurrently with radiation, limiting radiation dose, and use of minimally invasive surgery [37]. HPV-positive cells are believed to be more sensitive to radiation as compared to HPV-negative cells [37].

## 15. Conclusion

HPV-positive head and neck cancers thus differ from HPV-negative tumours in epidemiology, clinical features, molecular profile, and response to therapy. Due to this, identification of HPV positivity is essential in all cases for better management of the patient.

## Summary

Human Papilloma Virus associated oral and oropharyngeal head and neck carcinomas have features which are distinct from the tobacco and alcohol related squamous cell carcinomas.
